# Peptide-based functional annotation of carbohydrate-active enzymes by conserved unique peptide patterns (CUPP)

**DOI:** 10.1186/s13068-019-1436-5

**Published:** 2019-04-30

**Authors:** Kristian Barrett, Lene Lange

**Affiliations:** 1 Department of Biotechnology and Biomedicine, Technical University of Denmark, Kgs. Lyngby, Denmark; 2 BioEconomy, Research & Advisory, Valby, Denmark

**Keywords:** Peptide pattern recognition, Automated protein clustering, Protein group creation, Automated functional protein annotation, Systemized genome enzyme discovery

## Abstract

**Background:**

Insight into the function of carbohydrate-active enzymes is required to understand their biological role and industrial potential. There is a need for better use of the ample genomic data in order to enable selection of the most interesting proteins for further studies. The basis for elaborating a new approach to sequence analysis is the hypothesis that when using conserved peptide patterns to determine the similarities between proteins, the exact spacing between conserved adjacent amino acids in the proteins plays a prominent functional role. Thus, the objective of developing the method of conserved unique peptide patterns (CUPP) is to construct a peptide-based grouping and validate the method to provide evidence that CUPP captures function-related features of the individual carbohydrate-active enzymes (as defined by CAZy families). This approach facilitates grouping of enzymes at a level lower than protein families and/or subfamilies. A standardized, efficient, and robust approach to functional annotation of carbohydrate-active enzymes would support improved molecular insight into enzyme–substrate interaction.

**Results:**

A new nonalignment-based clustering and functional annotation tool was developed that uses conserved unique peptides patterns to perform automated clustering of proteins and formation of protein groups. A peptide-based model was constructed for each of these protein CUPP groups to be used to automatically annotate protein family, subfamily, and EC function of carbohydrate-active enzymes. CUPP prediction can annotate proteins (from any CAZy family) with high F-score to existing family (0.966), subfamily (0.961), and EC-function (0.843). The speed of the CUPP program was estimated and exemplified by prediction of the 504,017 nonredundant proteins of CAZy in less than four CPU hours.

**Conclusion:**

It was possible to construct an automated system for clustering proteins within families and use the resulting CUPP groups to directly build peptide-based models for genome annotation. The CUPP runtime, F-score, sensitivity, and precisions of family and subfamily annotations match or represent an improvement compared to state-of-the-art tools. The speed of the CUPP annotation is similar to the rapid DIAMOND annotation tool. CUPP facilitates automated annotation of full genome assemblies to any CAZy family.

**Electronic supplementary material:**

The online version of this article (10.1186/s13068-019-1436-5) contains supplementary material, which is available to authorized users.

## Background

Improved systematic and validated use of the overwhelming amount of genome sequencing data can open the way for increased biological insight. Several different methodological approaches, such as BLAST [1], CD-HIT [2], DIAMOND [3], HMM [4], PPR [5] and dbCAN [6, 7], and several types of multiple sequence alignments, e.g., MUSCLE, GBLOCK, DIALIGN, and MAFFT [8–11] have been developed and used over the last decades. Further, and most importantly, the vast knowledge about carbohydrate-active enzymes has been meticulously curated and made easily accessible to the scientific community by construction and updating the CAZy database [12]. The development of the conserved unique peptide patterns (CUPP) method is based on the principle of peptide pattern recognition [13], where peptide patterns conserved through evolution of efficient metabolic carbohydrate-active enzymes are captured. Furthermore, the CUPP method optimizes use of knowledge about the CAZy enzymes which have been characterized to EC function. It is hypothesized that all members of a CUPP group of proteins, which share the same conserved unique peptide patterns, have the same function (or share functional related features) as the characterized enzymes belonging to that CUPP group. The CUPP method shares with the new SACCHARIS method the conceptually important improved feature that the high number of characterized enzymes available can also be used for improved functional annotation of noncharacterized enzymes [14]. However, although the SACCHARIS method produces highly informative and automatically generated phylogenetic trees, the specific functional annotation (to EC number function) of each protein requires manual inspection. The CUPP method initially constructs a tree (based on peptide pattern similarities), but it also processes the information further: protein CUPP groups are automatically identified, and a peptide-based model for each CUPP group is constructed, which forms the basis for providing functional protein annotation. The outcome of the SACCHARIS method is phylogenetic trees for manual inspection, whereas CUPP continues to automatically form groups and create models of each group for rapid annotation of known or new proteins [14].

Developing the system of enzyme protein families (and for some families also subfamilies) of carbohydrate-active enzymes (CAZy.org) has been essential for understanding enzymatic biomass conversion in nature [12]. This knowledge has provided the backbone for development of optimized blends of enzymes for industrial biomass conversion [15–17]. However, so far only a minute part of the bacterial and fungal enzyme diversity has been exploited industrially [18]. The new bioeconomy will include enzyme conversion of a broad spectrum of biomasses (aquatic and terrestrial, and of plant, animal, algal, and fungal origins) converted into many new types of value-added products (food and feed, including gut health-promoting ingredients, biobased chemicals, and materials as well as fuels). Thus, new and improved enzymes (and enzyme blends) will be required to achieve this. Yet only a small fraction of carbohydrate-active enzymes has been biochemically characterized due to the extensive skills and laboratory facilities required. To optimize the efforts and systematically expand the necessary characterization, the candidate enzymes should be selected carefully. Improved bioinformatics tools can facilitate optimized utilization of the overwhelming amount of genome and metagenomes [19–22].

Carbohydrate-active enzymes have been divided into five classes of enzymes: Glycoside Hydrolases, Glycosyltransferases, Polysaccharide Lyases, Carbohydrate Esterases, and Auxiliary Activity enzymes. These classes have been further divided into protein families (CAZy.org). The glycoside hydrolases are the most intensively studied carbohydrate-active enzymes. However, only four families have been organized into subfamilies [12], GH5 [23], GH13 [24], GH30 [12], and GH43 [25]. Some of these subfamilies have been assigned (EC) functions and some subfamilies remain uncharacterized. The creation of both family and subfamily delineations are based on multiple alignments in combination with specific CAZy knowledge related to the enzyme proteins. The creation of subfamilies is a significant step forward for the research community easily and systematically to report scientific findings with reference to a category of closely related enzymes, a subfamily delineation, which is robust across time. However, several EC functions are often found within one protein family or even subfamily. The presence of multiple functions in a family or subfamily makes it desirable to subdivide into smaller groups, in order to capture differences in function-related features at a level lower than subfamily, i.e., creating groups that preferably include only one EC function.

Similar proteins can generally be assumed to share biological features [14]; however, even very different protein sequences may have the same enzyme function. Busk and Lange [13] suggested that specific, conserved peptide patterns may be the key to identifying proteins with such similar functions. Evolutionary pressure for fitness with regard to metabolizing substrates (for support of growth and reproduction) has led to specific parts/peptides of the protein (the parts most essential for the enzyme function in question) that are conserved. Therefore, the use of conserved peptides as a method of describing and comparing protein sequences includes the information of adjacent unique conserved amino acids. In the current work, this particular use is hypothesized to add an additional layer of information and thus obtain a more biologically relevant clustering and annotation. This methodological approach has also in part been used by cluster database at high identity with tolerance (CD-HIT) [2] and peptide pattern recognition (PPR) and utilizes the principle referred to as sliding window [5]. The sliding window gives CD-HIT and PPR their capability to handle a large number of proteins with relatively low computational requirements. In general, it is expected that highly similar protein sequences share enzymatic activity, and for this reason, one representative sequence may represent all protein members of the group. Using the protein-clustering tool CD-HIT, a large number of representative sequences have been identified, which might be further grouped. Notably, the CD-HIT method has been used in combination with PHI-BLAST and MUSCLE for incremental clustering [26]. Recently, dbCAN2 launched an annotation pipeline combining three state-of-the-art family annotation tools [7]: the HMMER3-driven dbCAN [4, 6], the BLAST-driven DIAMOND [3], and the PPR-based Hotpep [27], which uses few conserved peptides (up to 70) for each protein group. The idea is that the three tools combined (where a minimum of two out of the three agree on a prediction) increases accuracy of family annotation of CAZomes (the proteins of the proteome, which are carbohydrate-active enzymes). The F-score of the combined tools was reported to be 0.93, whereas each of the programs individually has an F-score of about 0.87 [7]. The performance of each of the tools was optimized on six CAZomes of the well-established organisms, which resulted in a stricter choice of parameters for Hotpep (compared to those previously applied) and lowered the rate of false discoveries [5, 7].

The CUPP program introduced here represents a new bioinformatic approach for using the nonalignment-based concept of PPR (patent application [13]). Here we describe, validate, and exemplify the CUPP protein clustering and functional annotation program. It is our hypothesis that grouping of proteins based on patterns of conserved unique peptides allows prediction of EC function of noncharacterized enzymes in all cases where CUPP group includes biochemically characterized enzyme(s). In short, the CUPP sequence analysis program described here attempts to create functionally relevant clusters of proteins that share a unique pattern of conserved peptides. It is such clusters that can enable annotation of a given query protein to a predicted family, subfamily, and EC function, or to automated annotation of the entire CAZome within a genome. In the CUPP program, the sensitivity of functional annotation of proteins is attempted to be improved by introduction of peptides containing ambiguous amino acids as this allows for detection of longer “motif” regions with a potentially less-conserved center region.

## Results

The description below of function, output, and performance of the CUPP classification and annotation is facilitated by choosing specific protein families as case studies. More specifically, GH30 was chosen based on the following criteria: (A) protein family with published and validated subfamily delineations, (B) protein family with multiple members of well-characterized enzyme proteins. Choosing families fulfilling both criteria A and B provides a basis for stringent validation and benchmarking of the CUPP F-score. The CUPP settings applied to the GH30 training set were used for clustering of all protein families in CAZy for construction of a peptide database (CUPP library). Another family that lived up to the criteria was GH5 which was used as an unsoiled dataset tested after the parameters were optimized. The application of the CUPP library was exemplified by CUPP prediction of 12 CAZomes or genomes.

### Selection of optimal CUPP parameters (ex GH30)

**Fig. 1:**
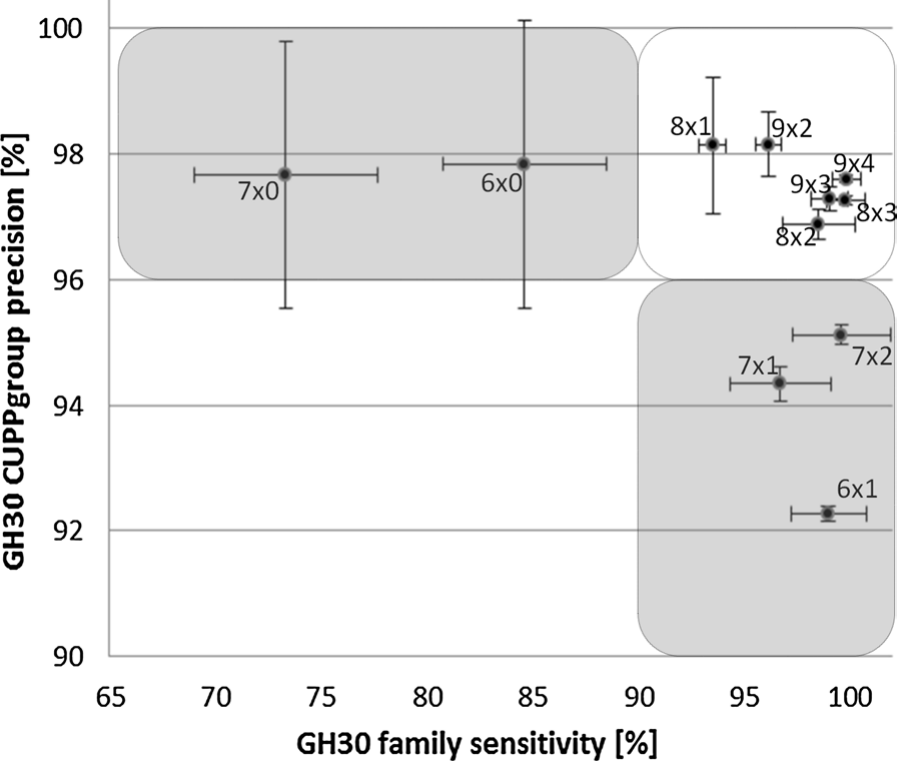
Selection of peptide parameters for CUPP clustering. The performance of CUPP clustering and prediction for GH30 using various peptide lengths and number of ambiguous amino acids within them (written as the length and the number of ambiguous amino acids separated by “x”). Each dot represents the average of CUPP group precision/family sensitivity for a c_clust ranging from 3 to 14 with corresponding error bars indicating the standard deviation. The gray areas indicate settings which are relatively less favorable. Peptides with a length of eight with two ambiguous amino acids were selected as the optimal for GH30 and applied for all CAZy families

The proteins of the GH30 protein family were clustered, and subfamily and function were predicted for each enzyme protein using a set of peptide parameters (length of peptide and number of ambiguous amino acids in the peptide) and clustering coefficient (c_clust) (Eq. 1 in “Methods”) in order to choose the optimal parameters (Fig. 1 and Additional file 1: Figure S1). The F-score was determined for subfamily and function over a range of clustering coefficient parameters (c_clust: from 3 to 14; Eq. 1), and the average and standard deviations of all results for each peptide parameter were determined (Fig. 1). A new algorithm for protein clustering, which benefits from peptides with insertion of ambiguous elements, results in improved precision and sensitivity. A peptide length of 8 with 2 ambiguous amino acids (8×2) was the optimal choice of peptide parameters (Fig. 1). The performance of parameters 8×3, 9×3 , and 9×4 was close to 8×2; however, the additional RAM requirements made them less favorable (Additional file 1: Table S1).

### Defining the unique peptides patterns for CAZy family GH30

Table 1 was constructed to obtain a summary of the individual CUPP groups within GH30 and their relation to subfamilies and EC functions. The number of different organismal taxonomic classes (bacteria, eukaryotes and fungi) represented within each group is indicated together with information of available PDB structures. Each CUPP group is expected to have some unique peptides (for the GH30 family up to 95%) among the peptides found in the unique peptide patterns (Table 1). Most GH30 subfamilies were divided into several CUPP groups and, notably, no CUPP groups of GH30 included members of more than one subfamily. Division of the GH30 family and subfamilies into CUPP groups separated the protein members into groups of proteins sharing the same conserved and unique peptide patterns (Table 1). The divergence among the proteins of GH30 was captured in 33 CUPP groups. The information regarding performance measured as sensitivity and precision of each CUPP group individually can be found in Additional file 1: Table S2.

**Table 1:** Summary of GH30 CUPP groups in relation to CAZy subfamilies and EC functions

GH30 Rep. members	CAZy classification			CUPP group	Number of peptides	Unique peptides	Taxonomy				Additional classification		
	Subfamily	Current	New				Classes	B	E	F	PDB	Function	Amount
11	GH30_1	20		GH30:1	2854	2488	4		X		24	3.2.1.45	2
9	GH30_1	20		GH30:2	4894	4098	6	X			1		
92	GH30_1	136		GH30:3	3349	2444	14	X	X				
29	GH30_1	33		GH30:4	11,750	11,306	4	X					
8	GH30_1	8		GH30:5	7182	6759	2		X				
45	GH30_2	101		GH30:6	1680	1450	11	X				3.2.1.37	3
5	GH30_2	9		GH30:7	7731	7571	1	X					
7	GH30_2	7		GH30:8	5334	4911	3	X					
22	GH30_3	35		GH30:9	7643	7007	4	X			2	3.2.1.75	1
8	GH30_3	12		GH30:10	12,014	11,253	3			X		3.2.1.75	4
85	GH30_3	90		GH30:11	3855	2991	10	X				3.2.1.75	1
30	GH30_3	54		GH30:12	9297	8260	1	X					
39	GH30_3	48		GH30:13	3279	1974	6	X					
31	GH30_3	45		GH30:14	1456	693	12	X				3.2.1.31	0
8	GH30_3	47	1	GH30:15	11,665	10,896	3	X					
8	GH30_3	15		GH30:16	8689	7905	3	X					
6	GH30_3	6		GH30:17	12,575	11,617	1	X					
5	GH30_3	2	8	GH30:18	8231	7565	2	X					
56	GH30_4	50	6	GH30:19	1331	892	10	X					
8	GH30_4	13		GH30:20	16,756	16,118	2	X				3.2.1.38	1
26	GH30_5	34		GH30:21	7515	7169	8	X		X		3.2.1.164	3
43	GH30_5	65		GH30:22	4914	4545	3	X					
12	GH30_5	12		GH30:23	2657	2091	6	X					
5	GH30_5	5		GH30:24	6845	6336	1	X					
6	GH30_6	5	2	GH30:25	3437	3315	2	X					
29	GH30_7	36		GH30:26	2524	2215	7	X		X		3.2.1.*	3
34	GH30_8	118		GH30:27	8402	7884	6	X			14	3.2.1.8&3.2.1.136	6
26	GH30_8	49	1	GH30:28	2923	2277	11	X	X		3	3.2.1.8&3.2.1.136	1
11	GH30_8	15		GH30:29	9608	9133	2	X					
13	GH30_9	25		GH30:30	11,596	10,861	2	X				3.2.1.31	1
5	GH30_		7	GH30:31	4096	3716	3	X					
7	GH30_		8	GH30:32	11,587	11,365	2	X					
5	GH30_		5	GH30:33	10,383	10,133	1	X					

Table 1 shows the available PDB structures and taxonomic statistics for the CUPP groups, which are listed along with the CAZy subfamilies and EC functions listed in CAZy. As shown, most families are subdivided by CUPP into several CUPP groups. Cases of bacteria and eukaryotes in the same CUPP group are found in five subfamilies (GH30:1, 3, 5, 7, and 8). The number stated in the column “Current” is the number of family domains found in proteins belonging to the given subfamily as delineated by CAZy. The column “GH30 Rep. Members” denotes the number of CD-HIT representative sequences in the given CUPP group, whereas the column “Current” indicates the number of proteins in the group, which have been assigned a subfamily by CAZy. (Note: if one protein represents several proteins of the same CD-HIT cluster, the subfamilies of these proteins also count). The “New” column refers to proteins in the groups with no current subfamily assigned. The column “Number of peptides” indicates the total number of peptides conserved among the proteins of the group, whereas the column “Unique peptides” indicates how many of these peptides are found only in the given CUPP group and not in any of the other CUPP groups of the family. The number in column “Classes” indicates the number of different organismal taxonomic classes represented in the individual enzyme CUPP group. B, E, and F correspond to the presence of members from bacteria, nonfungal eukaryotes, and fungi, respectively. The “&” character indicates multiple (here two) functions (EC numbers) found in the same entry in CAZy (or in the same CD-HIT High Similarity Cluster).

The GH30 family contains 1726 nonredundant and nonfragment proteins. 805 representative domain sequences were found by means of CD-HIT at 90%. A total of 734 proteins of these 805 sequences were assigned to a CUPP group. The remaining 71 proteins were removed either for being singletons or they did not have enough covered positions to be included in a CUPP group. The CUPP and dbCAN-HMM predictions of subfamilies were benchmarked against CAZy delineation (classification assigned by CAZy). The CUPP prediction of subfamily revealed a much higher performance compared to dbCAN-HMM. This was especially noticeable in GH30 subfamily 3 for which dbCAN-HMM had a sensitivity of 0.031 compared to a sensitivity of 0.993 for subfamily annotation by CUPP. Notably, the “fast-filtering” CUPP annotation F-score appears to be lower compared to “full-filtering” but is still superior to state-of-the-art tools. The CUPP annotation F-score of subfamily was overall at 0.992 (fast-filtering) or 0.996 (full-filtering), which indicates high performance (Table 2).

**Table 2:** Benchmarking of GH30 subfamily annotation

Sensitivity of GH30 subfamily annotation					Subfamily members
CAZy subfamily	dbCAN-HMM	dbCAN-Diamond	CUPP fast-filtering	CUPP full-filtering	
GH30_1	0.992	0.995	0.995	1.000	380
GH30_2	1.000	1.000	1.000	1.000	157
GH30_3	0.031	0.780	0.980	0.993	446
GH30_4	1.000	1.000	0.987	1.000	81
GH30_5	0.985	0.426	1.000	1.000	136
GH30_6	1.000	1.000	1.000	1.000	6
GH30_7	1.000	0.872	1.000	1.000	39
GH30_8	0.973	0.844	0.997	0.990	405
GH30_9	0.000	1.000	1.000	1.000	37
Overall	0.713	0.854	0.992	0.996	1687

As shown in Table 2, two settings of CUPP annotation (fast- or full-filtering) were compared to dbCAN-HMM and dbCAN-Diamond subfamily annotations. The sensitivity of the tools included in the dbCAN2 pipeline, able to perform subfamily annotation, is compared. Only proteins of GH30 having a subfamily delineation (classification assigned by CAZy) are included. The dbCAN-HMM database (release V7) does not have a model for subfamily 9 and the sensitivity was therefore recorded as zero.

**Fig. 2:**
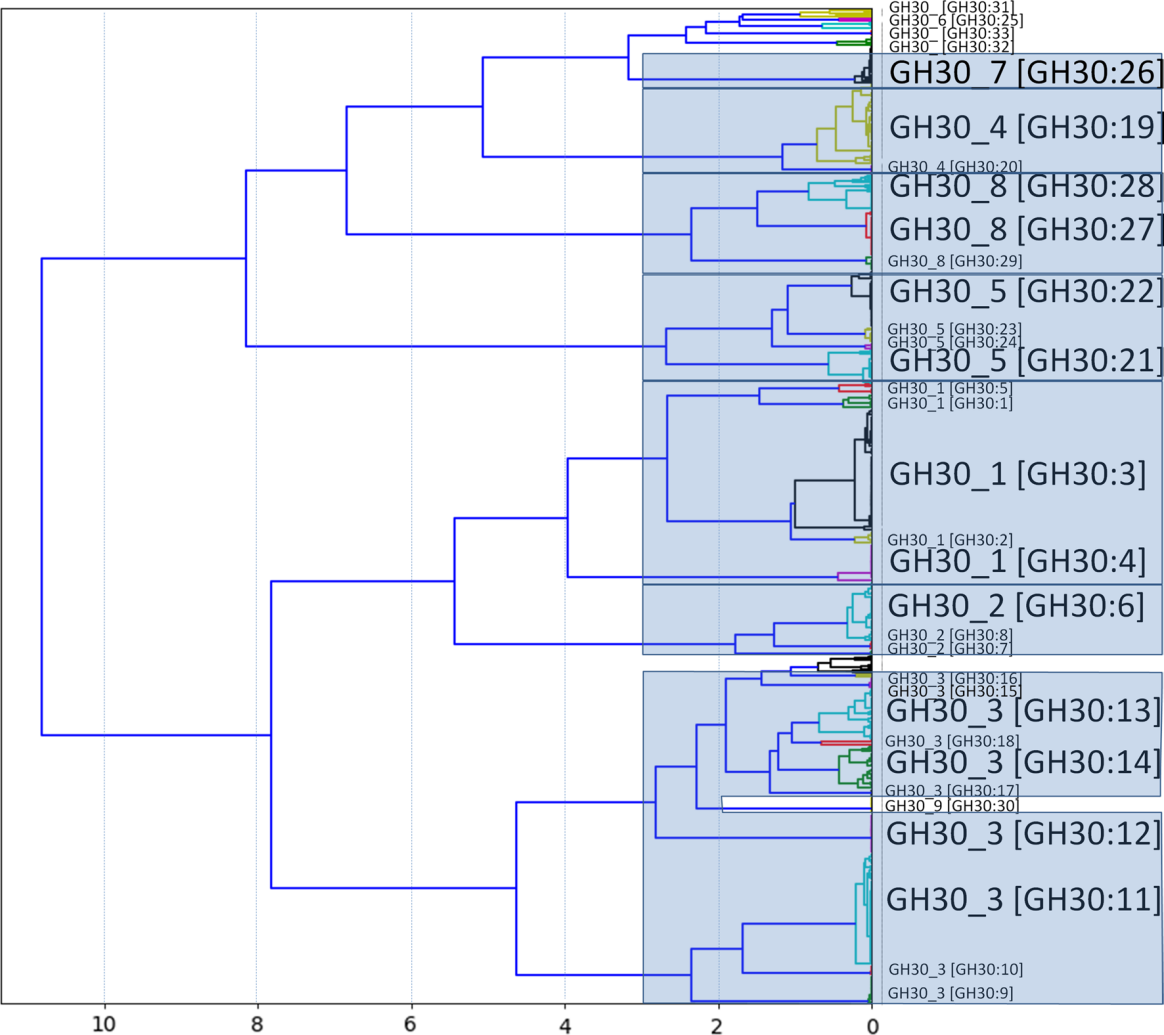
Dendrogram of the proteins involved in CUPP clustering of GH30. The 33 CUPP groups are indicated by labels. The distances on the x-axis are the “Ward” distances between the representative proteins belonging to GH30. The subfamily is designated with an underscore, whereas the content of the brackets are the CUPP group. Adjacent CUPP groups belonging to the same subfamily are indicated by blue boxes. The dendrogram was constructed as described in step 5 of CUPP clustering

The dendrogram in Fig. 2 was based on the conserved peptides the proteins share with each other. Construction of this dendrogram is described in Methods step 5 of CUPP clustering (see below). CUPP groups belonging to the same GH30 subfamily were placed by CUPP clustering as sister groups. However, the small GH30 subfamily 9 was located within GH30 subfamily 3, indicating a potential affiliation between GH30:30 and the functionally unknown GH30 CUPP groups numbered 13–18. Generally, all members of the same subfamily were found in a group below a threshold of three, and for GH30 subfamily 1 and 3 below a threshold of five, in the dendrogram (Fig. 2). This exemplifies the usefulness of the CUPP method for identifying subfamily affiliation based on dendrogram distances. However, manual assessment and CAZy validation and acceptance for formation of new subfamilies are still prerequisites.

### Comparing CUPP clustering with phylogenetic tree for CAZy family GH30

**Fig. 3:**
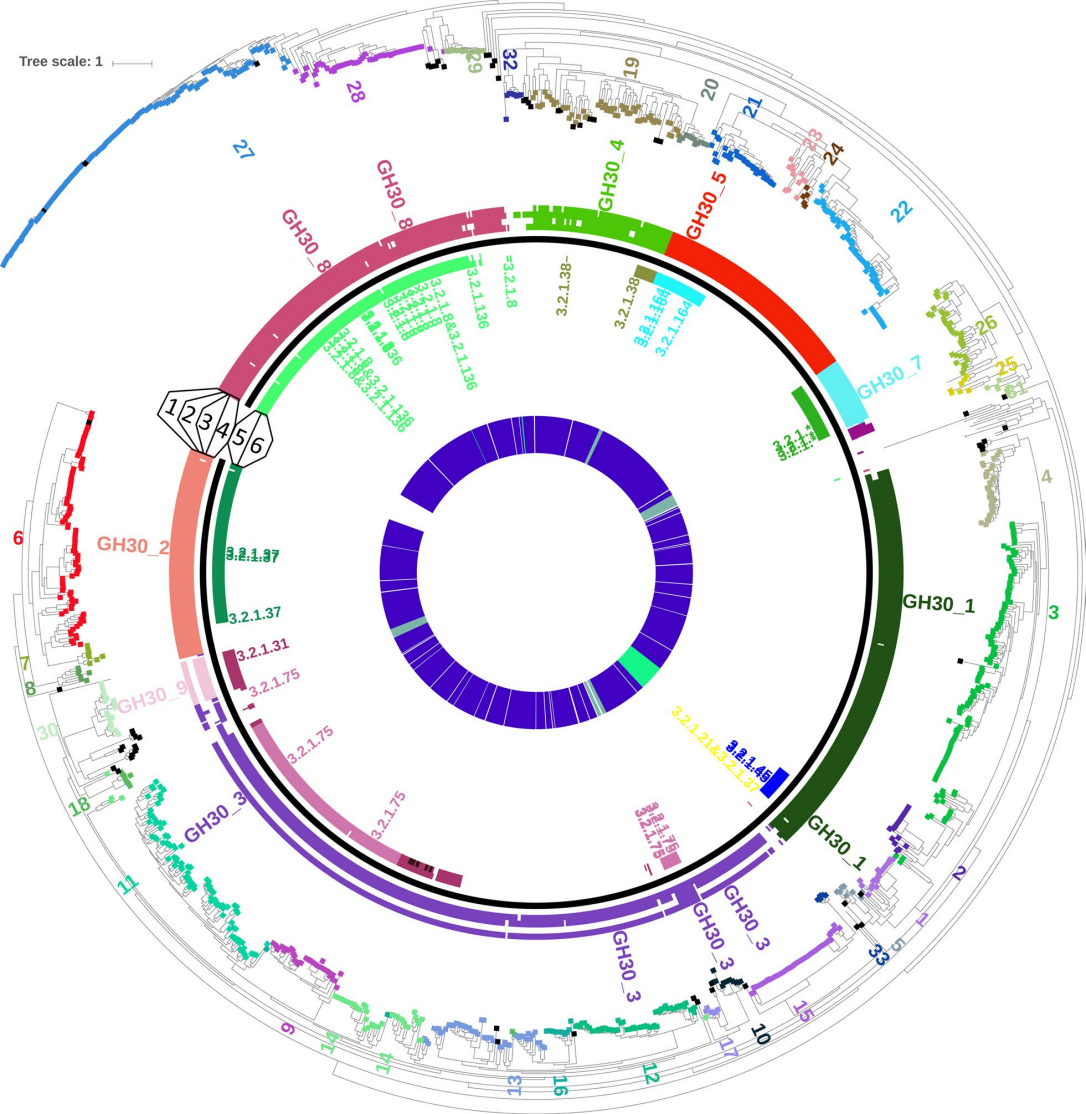
Inverted phylogenetic tree based on traditional multiple alignment of all GH30 protein domains. The numbering of the CUPP groups (numbered 1 to 33) is indicated directly on the tree. Each entry has a colored square indicating the CUPP group to which it belongs (black entries were ignored during CUPP clustering). Inside the tree are six numbered rings: the outermost ring, ring 1, indicates subfamily delineation according to CAZy and adjacent label of subfamilies; ring 2 is the subfamilies predicted by the dbCAN tool; and ring 3 and ring 4 show, respectively, the subfamily assigned by CUPP clustering and by CUPP prediction. Ring 5 and 6 represent the EC functions annotated by CUPP prediction for each entry as a result of CUPP clustering (ring 5) and of CUPP prediction (ring 6). White color (= empty spaces) in a ring indicates that no specific relation to subfamily or function could be assigned. The entries with functional annotation by CAZy are indicated by their respective EC numbers. The circles in the center indicates taxonomic groups where blue refers to bacterial, olive green refers to fungal, and lime refers to nonfungal eukaryote, while white refers to unknown taxonomy. An interactive version of the tree is available online: https://itol.embl.de/tree/1302256425066571528733948

All domains of GH30 predicted by dbCAN-HMM were used for creation of a phylogenetic tree and as the basis for forming the 33 CUPP groups of GH30, which are each identified by a number (see Fig. 3). The constructed tree was used to directly connect the subfamily delineation of CAZy to the predictions by dbCAN-HMM and CUPP for each individual entry. The protein members of the CUPP groups were generally found with short distances between one another in the phylogenetic tree (Fig. 3). However, based only on the tree, it would be difficult to manually determine exactly which proteins were members of which CUPP groups. For example, GH30:7 appears to be within CUPP group 6 and likewise CUPP group 20 appears to be within CUPP group 19. Higher resolution was achieved by constructing the dendrogram (see Fig. 2) in which similarity distances are based on peptides. This in itself exemplifies the enhanced separation achieved using peptide-based CUPP clustering.

The three entries with EC function originating from eukaryotes (EC 3.2.1.8, 3.2.1.75 and 3.2.1.21&3.2.1.37) were lost during CUPP clustering because the similarity to any one CUPP group was too low. However, during CUPP prediction, EC function 3.2.1.8 was still correctly annotated, and the two other functions were also annotated to the correct subfamily and to unknown function. This result indicates that CUPP clustering is robust even across broad taxonomic distances. As can be seen in Fig. 3, the grouping that results from CUPP clustering is generally in agreement with clusters manually identified on the phylogenetic tree. The consensus of subfamilies across CAZy, dbCAN-HMM, and CUPP clustering and CUPP prediction suggests that CUPP is a robust clustering and prediction tool. In the tree, the members of the individual CUPP groups were generally placed close to each other and often located in minor but dense branches of the phylogenetic tree. For the EC function assignment, there was also consensus between the information of CAZy and the information assigned by both CUPP clustering and CUPP prediction.

### Ability to predict new members of the family by CUPP (ex GH5)

A completely independent dataset was selected (family GH5) to enable full validation of the performance of CUPP clustering and CUPP prediction. This dataset had not been included in any training or optimization work connected with the development of the CUPP method. Family GH5 thus served as an unsoiled dataset to simulate the addition of new proteins to CAZy in the future before the model is updated to include them. The GH5 family was separated into two subsets, and one of these subsets (90% of the proteins) was used for CUPP clustering while the other (10% of the proteins) was used for CUPP prediction. The proteins of the two sets may have up to 70% sequence identity according to CD-HIT. The resulting observed sensitivity of CUPP annotation to family was 0.952, whereas the annotation to subfamily and EC function sensitivity scores were 0.975 and 0.925, respectively. The precisions of subfamily and functional prediction were 0.995 and 0.704, respectively.

### Performance of CUPP on the complete set of CAZy families

A CUPP library (database of conserved peptides) containing Auxiliary Activities (AA), Carbohydrate Esterases (CE), Polysaccharide Lyases (PL), Glycoside Hydrolases (GH) and Glycosyltransferases (GT) was created to elucidate further the robustness of the CUPP method across CAZy families when proteins of both closely and distantly related families are included. The CUPP clustering of all 306 CAZy families took 20 h on a single computer using eight cores without any need for manual inspection. In this run, however, eleven families (AA14, GH80, GH96, GH118, GH120, GH124, GT38, GT45, GT72, GT78, and GT97) could not even form a single CUPP group with the available sequences using the default settings. Instead, a reduced setting was applied for these eleven families. The reduced settings were as follows: the minimum number of protein members in a CUPP group was set to three; no CD-HIT and representative proteins were used only when proteins were identical; and no dbCAN-HMM predicted domains. The complete CUPP library v1.0.14 (306 CAZy families) was used on a FASTA file containing all proteins of all CAZy families combined. This resulted in F-scores for family, subfamily, and EC-functional prediction as 0.966, 0.961, and 0.843, respectively (Additional file 2). The CUPP library contains 6581 CUPP groups with 23,254,445 different peptides in total. In addition, dbCAN-HMM was used as benchmarking (using release V7 with an e-value cutoff at e-15 and a coverage of > 0.35) which resulted in F-scores for family and subfamily annotations of 0.956 and 0.950, respectively. Furthermore, the performance of CUPP for multimodular proteins had a slightly lower F-score (0.888) for prediction of proteins compared to single domain proteins. However, a similar reduction was observed for dbCAN-HMM (F-score 0.861).

The CUPP predictions of all 504’017 nonredundant proteins of CAZy (not including CBMs) took 3 h and 47 min using a single processor with full-filtering mode. The prediction within a few families was poor, and CE6, for example, had a sensitivity of only 0.15 (without dual domains, Additional file 2). However, in an effort to inspect the reason for the low CUPP performance on CE6, CUPP clustering using full-length proteins was attempted which resulted in a family sensitivity of 0.94. In these cases, inspection of the predicted domain ranges based on full-length proteins revealed that only a fraction of the domain is predicted by dbCAN-HMM and the majority of the conserved positions are outside the dbCAN-HMM predicted domain. Moreover, using CUPP clustering for GH22 gave a sensitivity of 0.79 while sensitivity with dbCAN-HMM was 0.82. However, by using full-length proteins instead of dbCAN-HMM predicted domains, the sensitivity of CUPP increased to 0.91. PL21 also had a low sensitivity of only 0.47, but using full-length proteins instead of the domains resulted in a sensitivity of 1. The CUPP model for AA7 had a sensitivity of 0.86, whereas the CUPP model of AA7 using full-length proteins gave a sensitivity of 0.93. Even though a significant increase can be achieved by using full-length proteins, the multimodular nature of proteins may cause issues. For example, an issue could occur in cases whereas frequently coexisting conserved domain is found in a protein family, and conserved peptides from both domains become mixed in one model. Thus, whenever possible, the domain regions are always used despite the potentially better performance of using full-length proteins.

### Benchmarking of CUPP performance for genome annotation

The performance of CUPP family prediction was compared to that of the three dbCAN2 tools in relation to the curated proteins of six CAZomes (see Table 3).

**Table 3:** F-score of CUPP prediction in relation to dbCAN2 CAZy family annotation tools

Species of CAZomes	CUPP F-score		dbCAN2 tools F-score				Relevant Proteins in CAZome
	CUPP fast-filtering	CUPP full-filtering	Predicted by ≥ 2 tools	dbCAN-HMM	dbCAN-Hotpep	dbCAN-Diamond	
*Arabidopsis thaliana*	99.39	98.76	99.23	97.70	97.71	96.52	980
*Aspergillus nidulans FGSC A4*	96.98	96.40	97.95	95.63	92.60	95.44	424
*Saccharomyces cerevisiae S288c*	98.58	98.58	99.80	98.58	99.80	97.33	91
Average of Eukaryote CAZomes	98.32	97.91	98.99	97.30	96.70	96.43	
*Caldicellulosiruptor bescii DSM 6725*	98.61	94.80	96.59	96.59	89.68	89.02	94
*Escherichia coli K-12 MG1655*	98.38	97.27	97.27	95.56	97.02	94.38	119
*Hungateiclostridium thermocellum ATCC 27405*	97.19	94.73	96.75	98.72	87.11	74.74	125
Average of bacterial CAZomes	98.06	95.60	96.87	96.96	91.27	86.05	
Average of CAZomes	98.19	96.76	97.93	97.13	93.99	91.24	
Complete runtime for the CAZomes [s]	34.33	54.23	255.23	128.61	91.25	35.37	

Table 3 shows the three CAZy family annotation tools of dbCAN2 that were benchmarked to the CUPP annotation using six CAZomes. The F-scores and runtimes are given individually for CUPP, dbCAN-HMM (database release V7), dbCAN-Hotpep, and dbCAN-Diamond. The three dbCAN2 tools can be combined to obtain a better prediction of which minimum two of the three tools need to agree on a family annotation (Predicted by ≥ 2 tools).

As shown in Table 3, the “full-filtering” mode of CUPP has very high precision for a minor loss of sensitivity, whereas the “fast-filtering” mode of CUPP results in higher sensitivity but also includes hits with lower support (achieved by omitting domain-filtering and domain-length requirements to give improved score values).

In addition to the performance of CUPP on only the CAZome fraction of the genomes, an additional comparison was conducted on the CAZome including the non-CAZome proteins which thus serves as a true negative dataset (Table 3). Based on the results given in Tables 3 and 4, we conclude CUPP to be a rapid and robust tool for genome annotation.

**Table 4:** F-score of CUPP prediction in relation to dbCAN2 CAZy family annotation tools including both CAZome and non-CAZome proteins

Species of genome origin	F-score of CUPP		F-score of dbCAN2 tools				Relevant proteins in CAZome	Proteins in genome (NCBI)
	CUPP fast-filtering	CUPP full-filtering	Predicted by ≥ 2 tools	dbCAN-HMM	dbCAN-Hotpep	dbCAN-Diamond		
*Botrytis cinerea B05.10*	95.77	95.44	96.7	95.59	87.5	94.18	341	13,703
*Malassezia restricta KCTC 27527*	95.33	95.38	96.1	92.56	84.89	95.23	80	4406
*Vigna angularis Jingnong6*	97.73	97.81	98.38	95.82	96.71	95.78	1133	37,769
Average for eukaryote genomes	96.28	96.21	97.06	94.66	89.7	95.06		
*Bifidobacterium bifidum NCTC13001*	97.36	96.48	95.11	90.19	93.2	82.99	59	1736
*Caulobacter segnis ATCC 21756*	97.94	97.09	97.26	96.21	91.89	97.97	115	4102
*Xanthomonas campestris ATCC 33913*	98.38	98.18	97.75	96.3	93.95	95.95	153	4179
Average for bacterial genomes	97.89	97.25	96.71	94.23	93.01	92.3		
Average of genomes	97.09	96.73	96.89	94.45	91.36	93.68		
Complete runtime of genomes [s]	785	808	6503	3375	2152	976		

Table 4 the three CAZyme family annotation tools of dbCAN2 were benchmarked to the CUPP annotation using six genomes (in addition to the 6 genomes analyzed in Table 3 including both CAZome and non-CAZome proteins). The F-scores including runtime are given individually for CUPP, dbCAN-HMM (database release 7), dbCAN-Hotpep, and dbCAN-Diamond. Furthermore, the combination of the three dbCAN2 tools was used to give a better prediction of which minimum two of the three tools needs to agree on a family annotation (Predicted by ≥ 2 tools). The dbCAN2 tools were run using default server settings [7]. The “full-filtering” mode of CUPP has very high precision for a minor loss of sensitivity, whereas the “fast-filtering” mode of CUPP results in higher sensitivity but also includes hits with lower support.

## Discussion

The combined CAZy research efforts, curation, and database maintenance and development, which cover protein family and subfamily definitions and delineations (including GH, GT, CE, PL, and AA proteins), are central to increased insight in carbohydrate-active enzymes and are valuable for the design of experimental work [12]. The CAZy system as such is recognized and widely used by the international scientific research community. Furthermore, the dbCAN2 analysis platform (now also including the genome annotation-optimized version of the PPR-based Hotpep) has been developed to be a state-of-the-art family prediction tool for carbohydrate-active enzymes [5–7]. However, an unmet need still remains for an even stronger, automated, and robust protein functional annotation tool that is suitable for the ever growing pool of genomic sequences. In this endeavor, the CUPP method represents a step forward. The CUPP method builds on the invaluable CAZy database (cazy.org) and the dbCAN-HMM prediction tools [6, 7]. To these tools CUPP adds additional value through capturing protein features which may be of relevance for function (viz. conserved unique peptide patterns) at a level below the protein family and subfamily. A high sensitivity of prediction using CUPP has been achieved by introducing ambiguous amino acids in the peptides, which allows the peptide units to be longer without making them too specific. The test runs reported hereon GH30 provide support for this conclusion.

A significant step in the validation of the CUPP method was made by using the N-fold cross validation approach in which a small part of the data is omitted from the training set and used as an unsoiled dataset [28] (Additional file 1: Figure S2 and Table S3). In the N-fold cross validation, the functional prediction is sensitive and does not forcibly assign a function to a protein but keeps them unknown. This makes CUPP reliable for in silico screening of genomes. Furthermore, validation of the CUPP method was achieved by constructing a single CUPP library with conserved unique peptides of all CAZy families and then using this library to determine the precision (here reported to be 0.999) among proteins included in the families. The high F-score for family and subfamily annotation is an indication of the robustness of the performance of CUPP annotation.

The results from the GH5 family study support the claim that the CUPP method is compatible with and fully capable of performing when used on sequence data and models that were not used in the training of the model, and in predicting proteins not included in the model. This test serves as a simulation of how well CUPP will perform on new proteins that are not identical to any of the proteins currently included in CAZy. However, if proteins with multiple functions (EC numbers) are placed in the same CUPP group, it may not be possible to tell whether one or the other is the most likely EC function of the query protein. However, when abundance is taken into consideration, a slight bias may be introduced toward the more well-studied EC functions, which might overshadow rarer EC functions within a CUPP group.

More specifically, in case two EC functions are found in the same CUPP group, the CUPP program can distinguish between the following two scenarios: In case, e.g., two EC numbers (3.2.1.4 and 3.2.1.21) are found in the same CUPP group, the functional assignment string can be written as 3.2.1.4 & 3.2.1.21 or as 3.2.1.4–3.2.1.21. The hyphen “-” between the two EC numbers indicates that the two functions are from distinct proteins, whereas the “&” indicates that the two EC functions are from the same protein (or from two very similar proteins (90% CD-HIT)). In the former hyphen-scenario (in order to avoid giving double functional assignment to single-function proteins), we combine two approaches: the EC function of the most abundant function is assigned to the query protein and the occurrence of the less-abundant function in the CUPP group in question is also informed. Then it is open for the user to trace such events.

In this first description of the new CUPP peptide-based protein annotation, we chose protein family GH30 (including subfamilies [29]) as a model case to describe the flow, use, and output of the CUPP method. As is shown here, the CUPP program appears to be able to match the state-of-the-art prediction tool dbCAN-HMM for prediction of families and subfamilies for carbohydrate-active enzymes. The GH30 family and subfamily prediction exhibited an F-score of 0.986 or above, a finding which supports the capabilities of the CUPP method. All proteins of GH30 were clustered when handled as one collection, and it was observed that all CUPP groups contained only one or no subfamily (Table 1). CUPP groups can contribute to facilitating subdivision of a subfamily or subdivision of families where no subfamily structure has yet been defined.

Surprisingly, for GH30 subfamily 3, the CUPP method identified 378 out of 381 nonredundant CAZy members in contrast to the 14 found by dbCAN-HMM (Table 2). This may be caused by the fact that the model for subfamily 3 available in dbCAN-HMM is from 2010 and is based only on 5 sequences [7]. The CUPP method could successfully annotate all proteins of the new GH30 subfamily 9 not included in release 7 of dbCAN-HMM. In the original paper, only 8 subfamilies of GH30 were reported [29]. In 2018, GH30 subfamily 9 was added to CAZy. However, the dbCAN-Diamond has been supplied with an updated database containing the members of subfamily 9, which enables prediction of this subfamily. The available subfamilies and EC functions are global for the protein, with no specification of which part of the protein is the responsible domain. This procedure may introduce noise into the prediction and reduce precision. However, this can be manually addressed, by altering or deleting the meta-data in the incorrect protein family based upon the results of a carefully conducted literature review. Such operations have not yet been conducted.

An interesting feature of the CUPP method is that it also provides a grouping of the part of the protein family where no members have been characterized. This facilitates the pinpointing of the types of proteins, which have a high level of novelty, as was exemplified by CUPP clustering of GH30 (see also Table 1, bottom). It also enables selection of members of each uncharacterized group for characterization, instead of having to screen every novel protein with no functional characterization. This feature is also an integrated part of the SACCHARIS program [14]. CUPP groupings as such can thus be used for guidance for intelligent selection of targets for enzyme discovery and for improved understanding of molecular interaction between microbes (or microbiome) and their substrate [30]. Notably, this also has relevance for the use of CUPP groups as lead for enzyme discovery, finding novel enzymes or finding new types of enzymes with specifically interesting functions of relevance for industrial application. A striking example concerns the case of the two entries of GH30 subfamily 8 with the same EC number (EGD48159.1 of CUPP group 27 and AAK76864.1 of CUPP group 28). In a recent study, St John et al. [29] described two proteins both belonging to GH30 subfamily 8 (AAK76864.1 and EGD48159.1), which have a dissimilar loop region. One of these proteins (EGD48159.1) requires α-1,2-linked glucuronic acid for hydrolyses, whereas the other (AAK76864.1) can hydrolyze linear xylan and has an increased rate of α-1,2-linked arabinofuranose substitutions [31]. This is an example of the ability of CUPP to capture differences in substrate specificities within this subfamily. Similarly, it was reported that peptide-based clustering of GH45 (by PPR) divided the protein into groups and captured differences in their 3D structure [32]. Biochemical activity testing also supported the distinction of these groups.

Inspection of the dendrogram (Fig. 2) shows that the recently created GH30 subfamily 9 is located within subfamily 3, and this connection also appears in the phylogenetic tree (Fig. 3). This could suggest that the new GH30 subfamily 9 is possibly a functionally diverse group within subfamily 3. Notably, the CUPP clustering could have been initiated by clustering each of the available subfamilies individually. However, by doing so we would have risked missing the inter-subfamily relations, e.g., in the case the relationship between subfamily 9 and some of the CUPP groups of subfamily 3. We chose to start with the whole protein family and to take a more holistic approach that will allow a wider use of CUPP clustering.

In this first published version of the CUPP program, we have validated CUPP for use for prediction of all CAZy protein families and not only families with published subfamily delineation (Fig. 2). CUPP clustering of protein families may also make it possible to include functional annotation for proteins not yet incorporated in the delineated subfamily structure [12, 25, 33]. Placing new proteins as members in the CUPP group structure may also lead to the tentative proposal of new subfamilies (Table 1). However, CUPP grouping based on conserved unique peptide patterns alone may not be sufficient input for delineation of new subfamilies. Confirmation by the CAZy expert validation and curation team will be needed for correct subfamily delineation that is robust overtime and acceptable to the research community.

From Table 1, based on information from 734 proteins, it appears to have been possible to capture the peptide pattern diversity of all the 1726 nonredundant GH30 proteins because almost all proteins were predicted correctly with an F-score of 0.993 (Additional file 2). The average F-score of a CAZy family (0.9657) was lower than that reported for GH30. Several examples have indicated that part of the issue may lie in the determination of the exact boundaries of the family domain region determined by dbCAN-HMM prior to CUPP clustering. The performance of the fraction of proteins having multimodular domains was lower (F-score 0.888) than reported for all CAZymes. However, the similar lower performance of dbCAN-HMM (F-score 0.861) indicates that both domains could be improved to cope better with multimodularity. Due to the outlier threshold of the program, it is possible to remove a small branch from a family in cases where a branch contains members that are both so different from other CUPP groups and also so diverse that the members cannot constitute a CUPP group on their own. One such group is present in GH30, located close to CUPP group 16 (Fig. 2). To include these proteins (by forming a new CUPP group), a second round of CUPP clustering should be performed with additional proteins similar to the lost/underrepresented proteins (found in the NCBI database). Alternatively, the “full-filtering” parameters during CUPP clustering could be reduced to allow formation of a smaller new group (3 or more members). For CUPP prediction, the default parameters are rather conservative in its annotation (named “full-filtering” in Tables 3 and 4). If there in specific cases is a need for also finding remote hits, the CUPP parameters can be relaxed (named “fast-filtering” in Tables 3 and 4). Fast-filtering, however, may introduce a few additional false positives since domain length and domain overlap are not considered to the same extent.

The F-score of CUPP prediction (“fast-filtering”) for the CAZome family annotation was one percent higher than that of dbCAN-HMM and far superior when benchmarked to dbCAN-Hotpep and dbCAN-Diamond. When the three tools included in dbCAN2 were used in combination (minimum of 2 tools agreed), the F-score was 0.979 which was just below the F-score of CUPP alone (0.982). When considering the runtime, CUPP prediction runs at about the same speed as the very fast dbCAN-Diamond tool and is seven times faster than the runtime of the three dbCAN2 tools combined. Notably, the CUPP library loading time, upfront, one time only, is not included in the time estimations. This omission is because the reason for measuring and improving speed is to be able to annotate millions of proteins in a short time, and in such cases, the initial loading time can be neglected. It should be mentioned that the datasets selected for this comparison were the CAZomes used for parameter optimizations of dbCAN-HMM, dbCAN-Hotpep, and dbCAN-Diamond. This may give those tools an advantage over CUPP which has not been trained on these specific CAZomes. The F-scores reported in the current work are much higher for all dbCAN2 tools than F-scores reported in the dbCAN2 paper, which is a result that was potentially caused by the removal of CBMs and a few new CAZy families (not included in release 6) [7]. An additional six CAZy-annotated genomes were selected for genomic annotation in the context of non-CAZyme proteins, and the result was almost the same overall F-score as reported for the CAZomes alone. However, the speed of the full-filtering mode of CUPP was increased to almost the same runtime as fast-filtering because the majority of the hits did not need any filtering. Moreover, when the runtime was compared to the tools individually, the speed of CUPP prediction exceeded the very fast dbCAN-Diamond [5].

CBMs were not included in the current work and not included in the CUPP library because they are considered to be a very different challenge as regards peptide-based annotation. The domain regions of CBMs are often small regions within much larger proteins, which makes clustering with full-length proteins complicated while the exact boundaries of the domain are difficult to determine. However, when a more curated data foundation (including exact boundaries of the domains) is available for all CBMs, they will be included in the CUPP library.

Measured in CPU, the computational requirements for running CUPP prediction are rather low. Though the RAM usage for holding models of all CAZy families is high (9 GB RAM), these models can still be accommodated on a modern laptop computer. It is noteworthy with regard to computational annotation of big volume protein data that multiple cores can be operated for both CUPP clustering and CUPP prediction, which makes the method more suitable for large scale usage. Generally, the high F-score obtained for CUPP prediction when compared to dbCAN-HMM in the various exemplifications (CAZome annotation, genome annotation, annotation of all CAZy proteins, and annotation of multimodular proteins for both family and subfamily predictions) establishes CUPP as a worthy new method for annotation.

If the protein family consists of more than 30,000 nonredundant/representative proteins, division of the family prior to CUPP clustering should be considered. This could be relevant for very large and complex families such as GT2 [14]. The main reason for large RAM usage during clustering is the construction of a distance matrix to determine the dissimilarities between each protein pair. This step consumes much computational power. Alternatively, it would be much faster to just choose one protein at random and start the clustering from there. Such an approach, however, is likely to introduce a bias that causes the first group to be inherently larger and thus reduce reproducibility and robustness. Such a clustering method has been applied to obtain the groups used by Hotpep [27] and some inconsistencies have been reported [34]. Though incremental clustering requires additional computational power, the distance between all proteins is considered for the CUPP clustering. The automated clustering approach, which is presented here and in the recent work by SACCHARIS, is an advantage for coping efficiently with the growing number of CAZymes in protein databases. Furthermore, harvesting synergy by new, integrated combinations of annotation tools could be achieved. CUPP prediction is capable of high performance alone. However, as shown by dbCAN2, a synergetic effect may be reached by combining several tools for even better performance.

Compared to Hotpep, we have improved the algorithm (and thus the CUPP method) in the following ways. For genome-based annotation, the CUPP prediction has a higher F-score and higher speed compared to the earlier peptide-based annotation tool Hotpep. The specific improvements are as follows: (1) The clustering of proteins for formation of the protein groups is based on five rounds of “all versus all” distance matrix to diminish the reported inherent bias toward the initial seed protein (forming the first group), which was reported to be greedy [34]. (2) Ambiguous amino acids have been introduced in the peptides because longer peptide lengths increased the overall sensitivity of the CUPP method. (3) CUPP identifies the conserved areas of the domain regions, which are used for filtering of the predicted domains for increased precision of the CUPP method. (4). Handling of very large datasets at high speed has been achieved by a single, upfront loading of all peptides for any number of FASTA files.

Furthermore, several features were added to improve usability of CUPP: (1) The approximate range of the domain is supplied in CUPP to give a better idea of the modularity of the protein. (2) CUPP can specify a query file or a folder of files and can operate on them directly as gzfiles (no need to unpack). (3) CUPP can also be used on bacterial genome DNA using a built-in ORF finder (beta version). (4) Among the CUPP outputs is a dendrogram that is converted into a Newick tree format together with label files to interact with iTOL (drag-and-drop) [35]. Regarding possible drawbacks, CUPP has a high RAM requirement for annotation, yet this requirement is still within the capacity of a modern laptop.

## Conclusion

Peptide-based classification was demonstrated to be successful for constructing automated protein groups each containing conserved unique peptide patterns. The conserved unique peptide patterns were also demonstrated to have enhanced capabilities for subfamily prediction compared to the state-of-the-art tool for subfamily annotation, dbCAN-HMM. Furthermore, the CUPP groups were used to automatically annotate carbohydrate-active enzymes to CUPP groups, protein family, and EC function. Evidence was provided (exemplified by CUPP prediction of GH30) that CUPP prediction can annotate proteins (from any CAZy family) with average F-scores for family, subfamily, and EC-functional predictions of 0.966, 0.961, and 0.843, respectively. The speed and F-score of CUPP were shown to match or improve on those of dbCAN2 tools, whether combined or individually, for both CAZy family and subfamily annotations. This achievement is based on the combined results of CAZome annotation, genome annotation, annotation of all CAZy proteins, and annotation of multimodular proteins for both family and subfamily predictions. The prediction was tested by N-fold cross validation in order also to work with proteins having high sequence divergence. CUPP facilitates automated annotation of full genome assemblies. A completely independent dataset, namely family GH5 which served as an unsoiled dataset, was selected to enable full validation of the performance of CUPP clustering and CUPP prediction. Family GH5 was separated into two partitions, and one part (= 90%) was used for CUPP clustering and the other part (= 10%) was used for CUPP prediction. The resulting family sensitivity observed was 0.952, whereas the subfamily and EC function sensitivity scores were 0.975 and 0.925, respectively. The precisions of subfamily and functional predictions were 0.995 and 0.704, respectively. The new CUPP method was validated through comparative genome-based annotation benchmarking of CUPP to dbCAN family prediction. This provides support for CUPP as a step forward toward peptide-based functional annotation directly from assembled genomic DNA. More analysis and validation are needed before the potential of CUPP for automated and efficient annotation of metagenomes can be assessed. The prediction of the 504,017 nonredundant proteins of CAZy in less than four CPU hours exemplifies the speed of the CUPP program. This result demonstrates that a standardized fast approach toward functional annotation of carbohydrate-active enzymes could facilitate advancement of molecular insight into enzyme–substrate interaction. Likewise, the CUPP program can be a valuable tool for guiding industrial enzyme discovery, optimizing discovery of new types of enzymes and finding more enzymes with a specific type of function.

## Methods

**Fig. 4:**
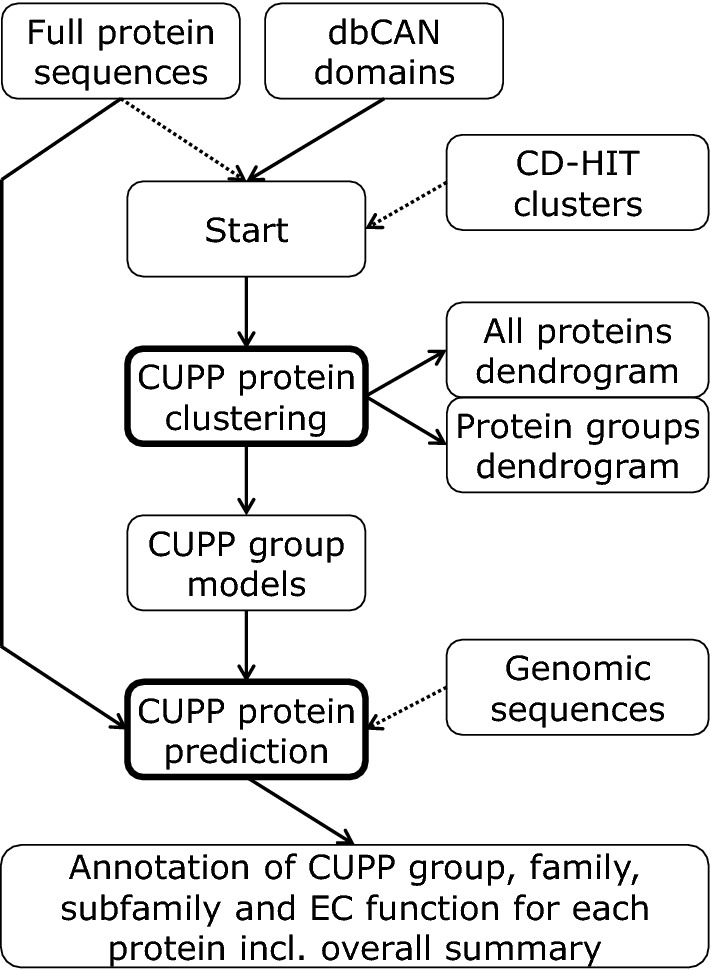
Flow diagram of the CUPP program. A CAZy protein family may be processed as a full-length protein or as the domain region alone (identified using dbCAN). A CD-HIT cluster file can be supplied which will reduce the number of protein sequences used in clustering by selecting one representative sequence for a cluster of highly similar sequences. The proteins are clustered based on conserved peptides, and for each of the resulting groups, a CUPP group is created consisting of the conserved peptides of the group. The distance between the individual proteins and the individual groups are saved as two separate dendrogram files (and distances between the proteins are also saved in Newick tree format for interaction with other programs). The CUPP group peptides are used in CUPP protein prediction for annotation of CUPP group to the query protein. In addition, the CUPP groups associated CAZy family, CAZy subfamily and EC function are also annotated to the query protein

The CUPP program consists of two separate parts. The first is responsible for clustering of proteins to create protein CUPP groups and obtain a peptide pool of conserved unique peptides for each CUPP group. The second part uses these peptide pools and associated meta-data to annotate proteins, e.g., from a genome assembly (Fig. 4).

### Representative proteins of family and data acquisition

NCBI GenBank accession numbers, CAZy enzyme family relationship, and function/EC numbers were downloaded from the CAZy database on 30th of April 2018. The corresponding protein sequences were obtained from NCBI GenBank along with NCBI taxonomy identifier. Since the actual domain region is not available, the family domains within each protein sequence were located using dbCAN-HMM prediction (database release 6) and filtering (hmmscan-parser.sh) with e-value cutoff e^−3^ [27]. Only domains of proteins listed by CAZy were included in the family collection [12]. Some proteins are overrepresented (many proteins from highly studied microorganisms) and dilute ·out the information of the underrepresented proteins (less studied species). To diminish this effect during clustering, a sequence was selected to represent multiple highly similar sequences by CD-HIT with a tolerance of 90%. This representative sequence received all the meta-data of all proteins of the high similarity cluster (identical meta-data strings of identical proteins do not count twice). Protein sequences listed as “fragments” by CAZy were not considered (Additional file 1: Figure S3). EC functions not stated in the “Activities in Family” field in the CAZy database are automatically removed from the individual entries for the target protein family.

### CUPP clustering

**Fig. 5:**
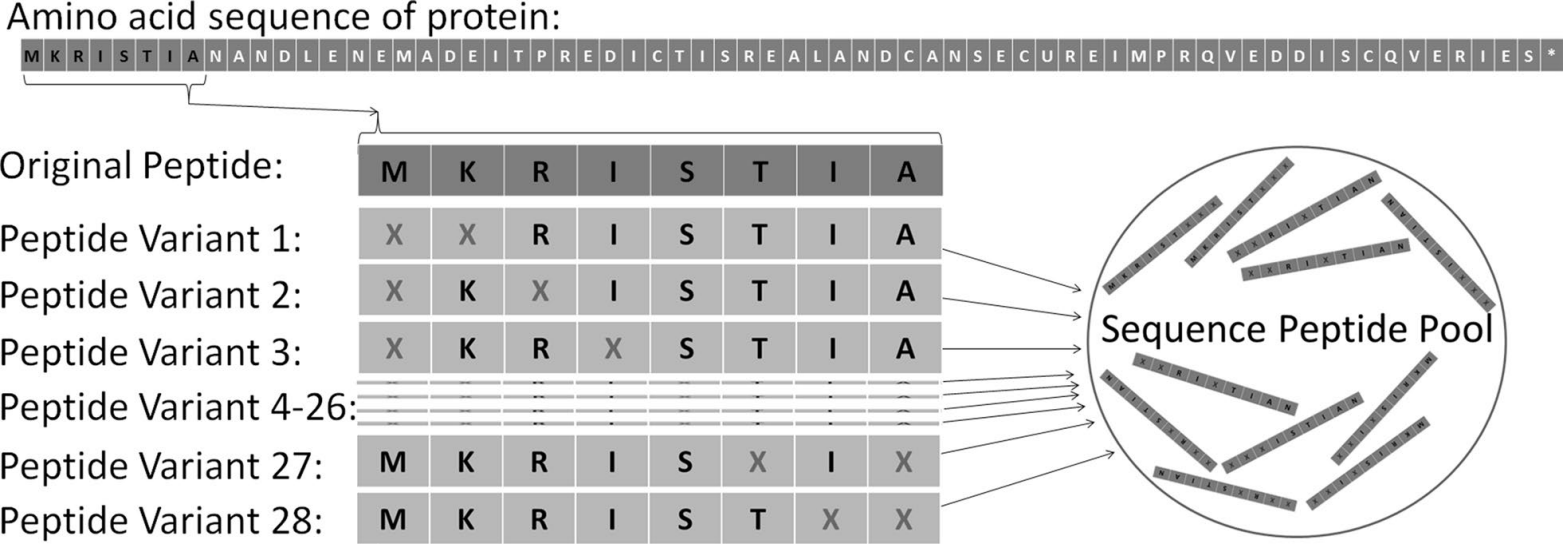
Formation of sequence peptide pool. The amino acids sequence of a protein is reduced to all peptides within a specified length of eight and exactly two ambiguous amino acids (In the peptides, ambiguous amino acids are indicated by an X). The window (indicated by the black letters) slides across the protein one amino acid at a time. For each position, “eight choose two” peptide variants are generated as strings and added to the sequence peptide pool. The black-colored letters of the protein sequence designate the first window (original peptide) from which the 28 peptide variants are created

The general concept of CUPP clustering is to transform the individual protein sequences into their peptides and thus obtain a sequence peptide pool (much like the bag-of-words model known from text mining where a page of text is represented only as the individual words) [36]. The sequence peptide pool is used to generate an index table of all peptides variants (as strings) found in each protein of a CAZy family. Every peptide shares the same predefined length (N) and number of ambiguous amino acids (A). Such peptides are constructed by a sliding window that moves across the protein in steps of one amino acid at a time [5]. For each original peptide of length N, all theoretical combinations of A ambiguous amino acids are generated to give “N choose A” combinations of peptide variants (Fig. 5). Insertion of ambiguous amino acids increases CUPP recognition of conserved peptides also when these have minor differences.

### Step-by-step description of the CUPP clustering

**Fig. 6:**
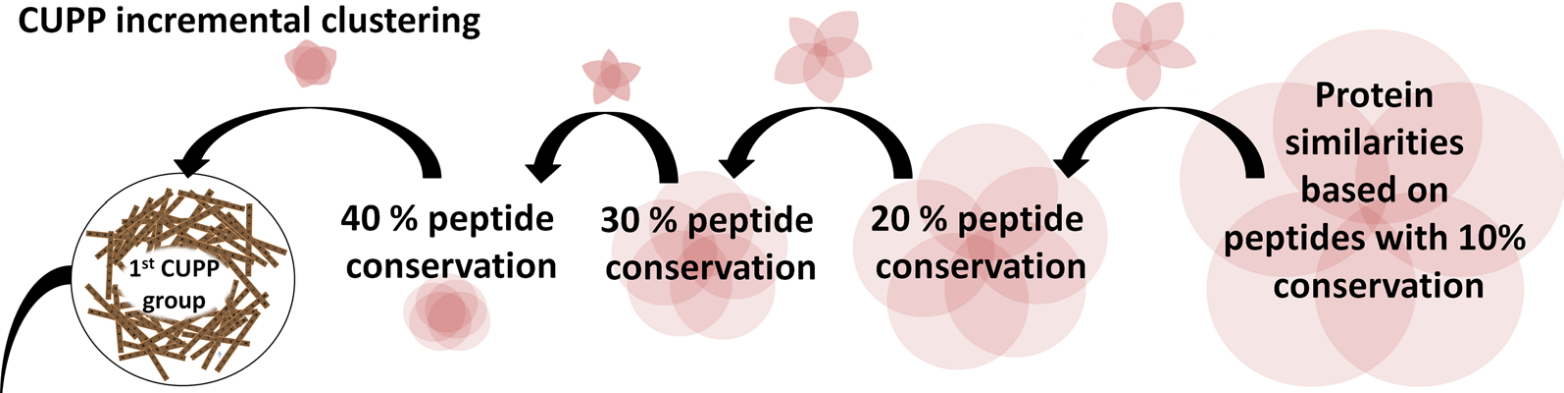
Flow diagram of incremental clustering indicating peptides shared between sequence peptide pools. Five-step incremental clustering where each red ring represents the set of peptides for a protein and the overlapping areas are peptides shared between two or more proteins. In the new iteration (indicated by the black arrow), only peptides exceeding a certain threshold of conservation are retained. Increasingly more conserved peptides were identified during five rounds of incremental clustering (illustrated as larger overlap between the rings of the Venn diagrams)

The final groups of proteins are obtained through multiple rounds of clustering, where the parameters are initially set loosely and increased for each of the iterations (referred to as incremental clustering). The purpose of the incremental clustering is to gradually amplify the signal of the conserved unique peptides and to use that signal to obtain protein groups that are likely to have similar enzyme function (Fig. 6). The CUPP clustering procedure is described in detail below (step 1–11):

1. A sequence peptide pool is created for each protein and includes all possible peptides along with the observed position in the original sequence.
2. Peptides found only in a single protein are removed from the sequence peptide pool. If any peptide is observed more than once in a single protein, that peptide is counted only once. In addition, during clustering (after the initial round), only peptides transferred from the previous round of clustering are kept.
3. Proteins having less than 20 positions covered by peptides (in cases where most peptides are removed in step 2) are considered outliers and disregarded for further clustering. In addition, the median of the number of covered positions for the proteins of the family is determined, and proteins having less than 10% (of the median) are considered outliers.
4. The distances between proteins are determined in a pairwise manner, and in this way, the positions covered by conserved peptides (not removed in step 2) are obtained for the two target proteins individually. The conserved peptides of the two target proteins are compared, and the shared conserved peptides between the two are identified (by exact string matching).
5. The pairwise distances calculated using Eq. 1 (see below) are used for construction of a distance matrix. The formed distance matrices are subjected to agglomerative hierarchical clustering using the linkage criteria “Ward” (Python package scipy.cluster.hierarchy.linkage) to obtain a linkage matrix. Flat clusters are formed from the linkage matrix for formation of protein groups using the criterion “Distance” with a threshold at 1 (Python package scipy.cluster.hierarchy.fcluster). The linkages can be directly visualized as a dendrogram (see Fig. 2).
  1 $$Score_{ij} = \left( {1 - \frac{shared\_positions}{2 \cdot max\_positions}} \right)^{c\_clust}$$
  Eq. 1—the conserved peptides of the two targets proteins are compared, and the shared peptides are recorded along with the first position of each peptide (start positions). To calculate the score, the numbers of different start positions of the shared peptides in both proteins combined are determined (shared_positions). The number of conserved peptide start positions in each of the target proteins individually is determined, and the maximum value of the two is used to calculate the score (max_positions). The user-defined (default 9) c_clust (clustering coefficient) is a positive integer and is used to obtain the desired signal amplification. in general, the greater the value, the fewer the CUPP groups will be formed. The number of conserved peptides decreases for each of the iterations during incremental clustering, which forces the dissimilarity closer to zero by reducing the denominator due to fewer conserved peptides (see illustration, Fig. 7).

**Fig. 7:**
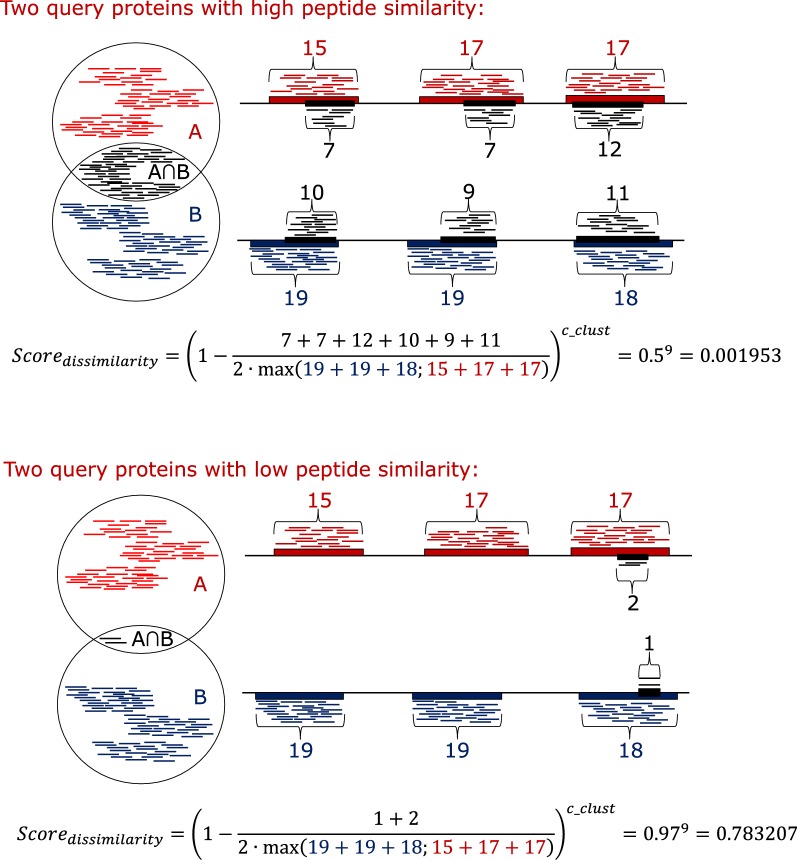
Exemplification of the CUPP clustering dissimilarity score. Determination of the dissimilarity score between two protein domain regions during CUPP clustering exemplified as two scenarios: one of high similarity between the two proteins (A and B) and one with a low similarity. The thick black horizontal line represents the amino acid sequence of the two target proteins. The conserved peptides found in protein A are indicated individually by the short red line above the protein. Similarly, beneath protein B, the presence of conserved peptides of protein B is shown as short blue lines. The subset of conserved peptides found in both protein A and protein B is represented by the short black lines between the two proteins. To determine the dissimilarity, the number of different positions covered by the conserved peptides is determined for each of the three colors of peptides indicated by the numbers (blue, red, and black). The dissimilarity score equals one minus the number of black peptide start positions divided by two times the maximum of the number of peptide start positions of the red or the blue peptides
6. Proteins placed in a group having only one member (during the initial round the minimum protein group size = 2) are ignored, and the remaining groups are further assessed.
7. For each of the protein groups, peptides found among a minimum of 10% of the proteins (during the initial round the peptide conservation = 10%) are included as conserved peptides of the protein group along with their peptide conservation (the conservation corresponds to the abundance of target peptide among the protein members of the group).
8. The created protein groups each have their own conserved unique peptides, although some of the individual peptides might be shared with a sister group. However, in cases where the peptides (shared by the two protein groups) are also the most conserved peptides in each of the groups, the separation of the two groups needs to be re-evaluated. To do this, the abundance of each peptide in each of the group is determined (peptide conservation). To determine the dissimilarity between the groups, an X times X distance matrix is created, where X is the number of groups. Each group is compared in a pairwise manner, and two measures are calculated: (1) the sum of the peptide conservation of the shared peptides between two target protein groups is determined (shared_conservation); (2) the peptide conservation of the peptides found in each of the two target protein groups individually is obtained, and the maximum value of the two is determined (individual_conservation). The dissimilarity between two target groups is defined as one minus the shared_conservation divided by the individual_conservation. This distance matrix is subjected to agglomerative hierarchical clustering using the “Complete” linkage criteria. Flat clusters are formed from the resulting linkage matrix using the criterion “Distance” set to have threshold at 0.7.
9. Steps 2–8 are repeated three times, and for each iteration, the minimum protein group size is increased by one, and the peptide conservation is increased by 10% (Fig. 5). However, for each new iteration, only the peptides considered conserved in step 7 of the previous iteration are included, whereas those not considered conserved are ignored. This removal happens in the same way as for peptides found only in a single protein during the step 2. This repeated operation (incremental clustering) proceeds until 40% conserved peptides are obtained.
10. Finally, peptides having a conservation of 40% are employed in a last round of clustering to obtain the unique conserved peptide pattern to be used to characterize the final protein groups (minimum default size 5); these groups are called CUPP groups.
11. For each of the resulting CUPP groups, the peptide conservation of each peptide is determined, and those above 20% are retained. To favor the more conserved peptides, the peptide conservation of each peptide is squared (e.g., a peptide conservation of 0.2 will be reduced to 0.04, whereas a peptide conservation of 0.9 will be reduced to 0.81). In addition, for later annotation, the meta-data associated with the included proteins of the CUPP groups is remembered.

### CUPP library construction

**Fig. 8:**
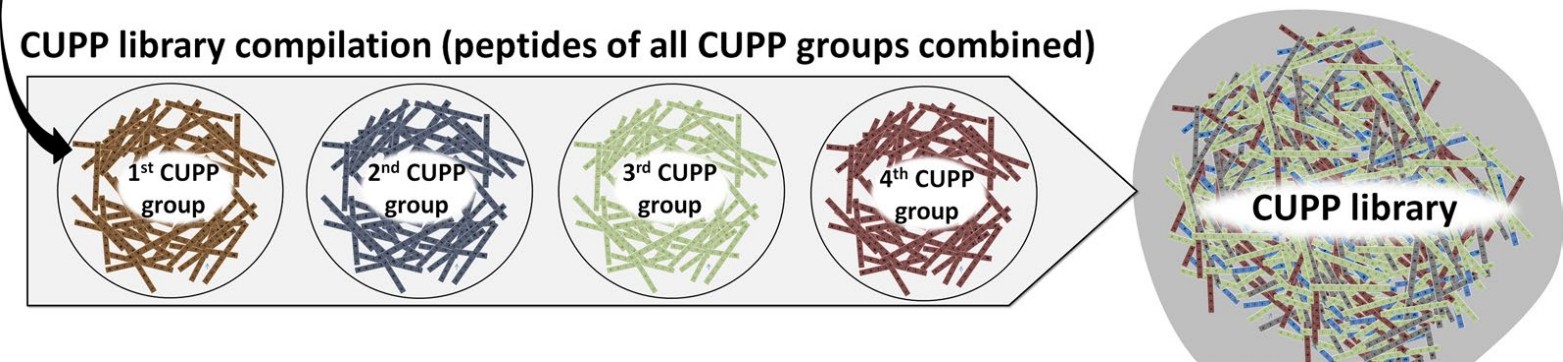
Compilation of the CUPP library in which CUPP group peptides are merged. The peptides of CUPP groups from all CAZy protein families were compiled into one CUPP library that was used as a look-up dictionary facilitating faster protein annotation

As described above, the CUPP clustering was constructed for each of the CAZy enzyme families. For each family the conserved, unique peptide patterns were identified which define the peptides of the CUPP group. A common index table referred to as a CUPP library is created to enable CUPP prediction of genomes without the need to inspect models of each individual family separately. Each peptide in the CUPP library along with its peptide conservation is associated with the CUPP group in which it is found. During compilation of a CUPP library, some peptides might be found in several protein families. When this is the case, the peptide conservation of such peptides is reduced in proportion to the number of families in which they occur (Fig. 8). In addition, peptides consisting solely of the abundant aliphatic amino acids or proline are banned from the CUPP library to avoid a bias toward linkers and polyproline regions [37].

### CUPP group prediction

Each protein is processed individually as described below (see also flow diagram—Additional file 1: Figure S3):

1. All peptides of the query sequence are obtained using the sliding window principle, including ambiguous amino acids, as previously described (Fig. 4). Each peptide found in the CUPP library is recorded along with its associated CUPP group name and its peptide conservation.
2. Two measures are calculated to determine if any CUPP group is found to be associated with the query protein. First, the sum of peptide conservation of the peptides shared between the query protein sequence and the CUPP group peptides needs to be at least five. Second, the sum of peptide conservation needs to be at least one percent of the theoretical maximum sum of peptide conservation of the peptides in the given CUPP group. In cases were one peptide is found twice in the same protein, it will count only once during the initial filtering. For fast-filtering, steps 3, 4, and 5 are left out, but peptides from eight different positions must always be present. For full-filtering, all steps are included in the analysis.
3. Each peptide associated with a given CUPP group is mapped to the protein sequence. The positions covered by exact string matching receive a position-specific score, corresponding to the peptide conservation of the current peptide. This results in a list of equal length to that of the protein sequence, and each position is the sum of peptide conservations of covering peptides (referred to as the list of accumulated peptide conservations). At least 20 positions (minimum domain length) of the list of accumulated peptide conservations need to be above 0.2 to be a valid prediction using full-filtering mode. For range determination, peptides found more than once count equally to detect, e.g., tandem repeats of exactly identical domains that are present in a single protein.
4. If more than one CUPP group remains, the covered lists of accumulated peptide conservation are inspected (by the program) for each group to see if they overlap. The group having the highest percentage of the theoretically maximum sum of peptide conservation is processed first and will be assigned to the protein. The covered positions of the first domain are recorded in a list of occupied positions along with the value of the position. A potential second domain will be assigned to the protein in cases where at least 50% of the sum of peptide conservation of the new domain is not already occupied by previously assigned domains.
5. The approximate range of each CUPP group is determined by inspection of the list (explained in step 3). Gaps below a threshold are considered as the same domain, whereas gaps larger than the threshold are considered as two separate domains (indicated by two ranges, e.g., GH30:1.1 (score, 90.0.190; 400.0.500)). The threshold is determined by the average number of included positions in the CUPP group (recorded during the final round of clustering) with a minimum of 50 and maximum of 200 amino acids.
6. The query protein will be annotated to the CUPP group(s) found. In addition, the query protein is assigned to the associated CAZy family, CAZy subfamily, and EC function of the predicted CUPP group(s). EC function and CUPP group are only assigned during full-filtering mode, whereas for fast-filtering, the general double zero-group will be assigned, e.g., GH30.0.0. However, for the query protein to be assigned to a subfamily, a CUPP group needs to have at least three members of the same subfamily. The full-filtering mode of CUPP prediction will only assign the predicted CUPP group (and the EC function of the CUPP group) to a query protein if at least five percent of the theoretical maximum sum of peptide conservation of the given CUPP group is achieved. If this criterion cannot be fulfilled, the protein will be assigned to a CUPP group zero dot one which indicates that the protein belongs in the family but not in any of the current CUPP groups (e.g., GH30.0.1). Furthermore, if two CUPP groups are assigned to a protein in the same range, the domain is denoted zero dot two for simplicity (e.g., GH30.0.2) (this simplifying operation can be overwritten by beta option “complex”).

### CUPP validation and benchmarking

N-fold cross validation was conducted by dividing the GH30 CAZy family into ten parts and using nine parts for clustering and one part for prediction to initiate 10% new proteins (repeating ten times until all parts have been left out). The proteins of the ten partitions may have up to 70% CD-HIT sequence identity. Each partition was created using CD-HIT clustering of 70% and adding one high similarity protein cluster at a time until a minimum of 10% of the total proteins was found in the partition, or until all proteins were distributed (starting with the largest cluster). For N-fold cross validation, prediction counts as correct in the cases where all similar EC functions are removed and the function is predicted to be unknown.

Online Multiple Alignment using a Fast Fourier Transform (MAFFT) server was used for multiple sequence alignment [38]. A phylogenetic tree was created from the multiple alignments using CIPRES and the RAxMLblackbox model with substitution matrix LG [39]. The resulting tree was further treated with labels using the Interactive Tree of Life (iTOL) web server for graphical interface [35]. For genome comparison, the dbCAN2 webserver was used with default settings for dbCAN-HMM, dbCAN-Diamond, and dbCAN-Hotpep [7]. The runtime calculations for dbCAN-HMM, dbCAN-Diamond, dbCAN-Hotpep, and CUPP were set up in a Linux environment using an Intel^®^ Xeon^®^ CPU E5-1660 v4 @ 3.2 GHz computer. Families not included in the 6th release of dbCAN or not included in CUPP (namely AA0, AA14, AA15, CE0, CE10, GH0, GH146, GH147, GH148, GH149, GH150, GH151, GH152, GH153, GT0, GT105, GT106, PL0, PL28, cohesion, and SLH) were ignored for all benchmark tools along with CBMs. HMMER3 software was used with dbCAN-HMM release V6 for determination of domains used for CUPP clustering. However, for benchmarking of family and subfamily annotation of GH30 and CAZomes annotation, the newly released V7 was applied [4, 12, 40].

For CAZome annotation, the protein sequences of the accession numbers listed in the CAZy database for the respective strains were downloaded from NCBI. For six genomes (including the CAZome annotations), the protein sequences were merged with the protein sequences of the respective strain from the NCBI assembly protein list. However, the accession numbers did not match. To achieve merging, CD-HIT clustering with a similarity of 99% was used. All proteins of a group having a CAZyme were assigned to its CAZy family, and the protein from the CAZome was deleted. The protein family named CE10 was ignored from dbCAN predictions, since CAZy no longer supports this delineation. For GH5, the family was divided into two datasets, and one part was used for clustering (90%) and the other part was used for prediction (10%) in the same way as the first partition of N-fold cross validation of GH30. Sensitivity is defined by the number of true positives divided by the number of total CAZy families found in the protein. Precision is defined as the number of true positives divided by the sum of true positives and false positives. The F-score is defined as the two times precision times sensitivity divided by the sum of precision and sensitivity.

## Additional files

**Additional file 1: Figure S1.** Selection of c_clust and peptide parameters. **Figure S2.** N-fold cross validation of GH30. **Figure S3** CUPP flowchart**. Table S1** Relative RAM requirements as a function of peptide length and number of ambiguos positions. **Table S2** GH30 CUPP group validation. **Table S3**. N-fold cross validation of GH30 using ten partitions.

**Additional file 2.** Performance of CUPP prediction for each CAZy family.

## Abbreviations

A: the number of ambiguous amino acids present in each peptides of a sequence peptide pool
AA: Auxiliary Activities
c_clust: clustering coefficient used to increase the similarity between each pair of proteins based on the peptides they share of the respective sequence peptide pools
CAZy: carbohydrate-active enzymes
CAZyme: carbohydrate active enzymes found in the CAZy database
CAZome: carbohydrate-active enzymes among the proteins found in a genome assembly
CBM: carbohydrate-binding module
CPU: central processing unit
CD-HIT: cluster database at high identity with tolerance
CE: carbohydrate esterases
CIPRES: cyberinfrastructure for phylogenetic research
CUPP: conserved unique peptide patterns
CUPP groups: a group of proteins sharing conserved unique peptides which can be used as markers for identification
CUPP library: all peptides considered to be important for any CUPP group used for CUPP prediction
dbCAN: DataBase for automated Carbohydrate-active enzyme Annotation
GH: glycoside hydrolases
GT: glycosyltransferases
HMM: Hidden Markov Model
Hotpep: Homology ToPEptide Pattern
iTOL: Interactive Tree Of Life
MAFFT: Online Multiple Alignment using Fast Fourier Transform
MUSCLE: MUltiple Sequence Comparison by Log-Expectation
N: length of each peptides in a sequence peptide pool
NCBI: National Center for Biotechnology Information
PL: Polysaccharide Lyases
RAxML: Randomized Axelerated Maximum Likelihood
