# Supplementary material for: Peptide-based functional annotation of carbohydrate-active enzymes by conserved unique peptide patterns (CUPP)
Source: Biotechnol Biofuels. 2019 Apr 30;12:102. doi: 10.1186/s13068-019-1436-5 (PMC6489277; doi:10.1186/s13068-019-1436-5)
Supplement: Supplementary file 1 — Additional file 1: Figure S1. Selection of c_clust and peptide parameters. Figure S2. N-fold cross validation of GH30. Figure S3 CUPP flowchart. Table S1 Relative RAM requirements as a function of peptide length and number of ambiguos positions. Table S2 GH30 CUPP group validation. Table S3. N-fold cross validation of GH30 using ten partitions. [file 13068_2019_1436_MOESM1_ESM.docx]

**Additional file for “Peptide-Based Functional Annotation of Carbohydrate-Active Enzymes by Conserved Unique Peptide Patterns (CUPP)” by Barrett & Lange**

**Figures S1 – S4:**

**Figure S1 Selection of c_clust and peptide parameters**
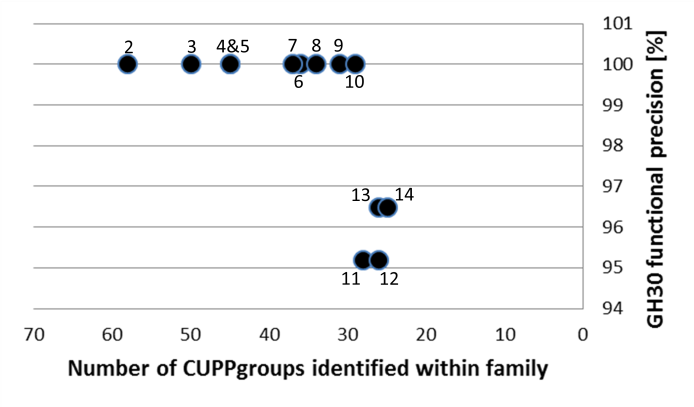


Figure S1 The impact of c_clust on the number of identified CUPP groups within the GH30 family vs the functional precision. The numbers on top of the dots indicate the c_clust coefficient. There is a switch after a c_clust of 10 where the precision drops by 4%, indicating too few CUPP groups were identified to predict the function precisely. When the c_clust is too high, the number of CUPP groups becomes limited and if two groups are squeezed together; one or the other will be lost. This may cause a CUPP group (in this case with an EC number) to be removed for a few rounds and later it may return, hereby causing an increase in the precision of the recovered EC function(s). However when the precision begin to fluctuate, c_clust should not be further increased.

**Figure S2 N-fold cross validation of GH30**


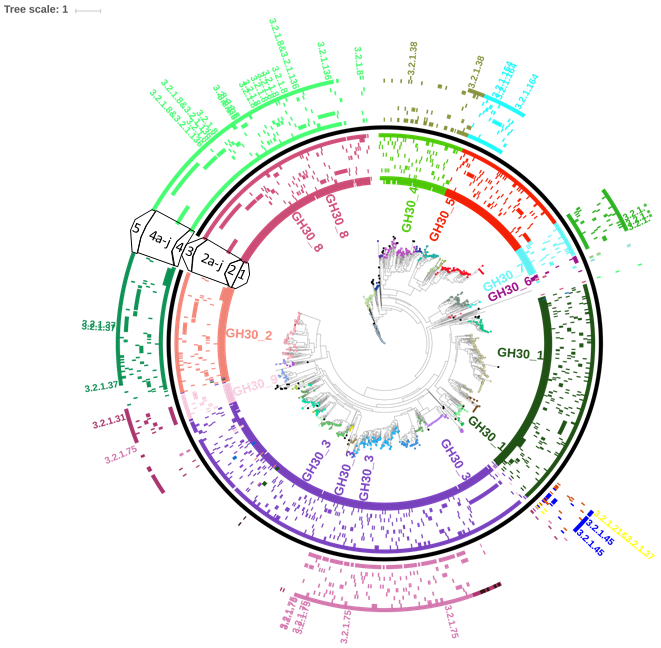


**Figure S2** N-fold cross validation of functional prediction of GH30 for ten rounds of 10% subsets. Ring 1 represents the subfamilies delineated by CAZy with a label of the same color. Ring 2 results from the ten rounds of N-fold cross validation merged into a single ring. The ten preceding rings represent each of the partitions of the N-fold cross validation shown individually (2a-j). Ring 3 is the subfamily prediction using the full collection for both CUPP clustering and prediction. The ring just outside the black (ring 4) is the merged functional prediction of the ten partitions of N-fold cross validation also shown individually (4a-j). The outer ring (ring 5) is the functional prediction of the full collection. The outer labels mark the entries holding an EC function recognized by CAZy. White indicates that no function was found or that the protein was included in clustering and therefore was not in the prediction. The “&” character indicates one protein has two EC numbers (in the case of 3.2.1.8&3.2.1.136, the two EC numbers are counted represented as the same color). Each surrounding ring was the result of a round of CUPP clustering of 90% of the proteins and the subsequent prediction of the 10% of proteins omitted. The N-fold cross validation of GH30 demonstrated the performance of CUPP prediction on a set of proteins not included in the model itself. The consistency of both subfamily and function prediction across the ten rounds of N-fold cross validation, supports that the method is capable of correct annotation of new proteins not included in the clustering. When an entry with an EC number is removed, the protein when predicted should not be assigned with another function but should either keep the same function or become of unknown function. For EC function annotation one percent of the maximum peptide sum in a CUPP group was required in contrast to the five percent used as default.

**Figure S3 CUPP flowchart**


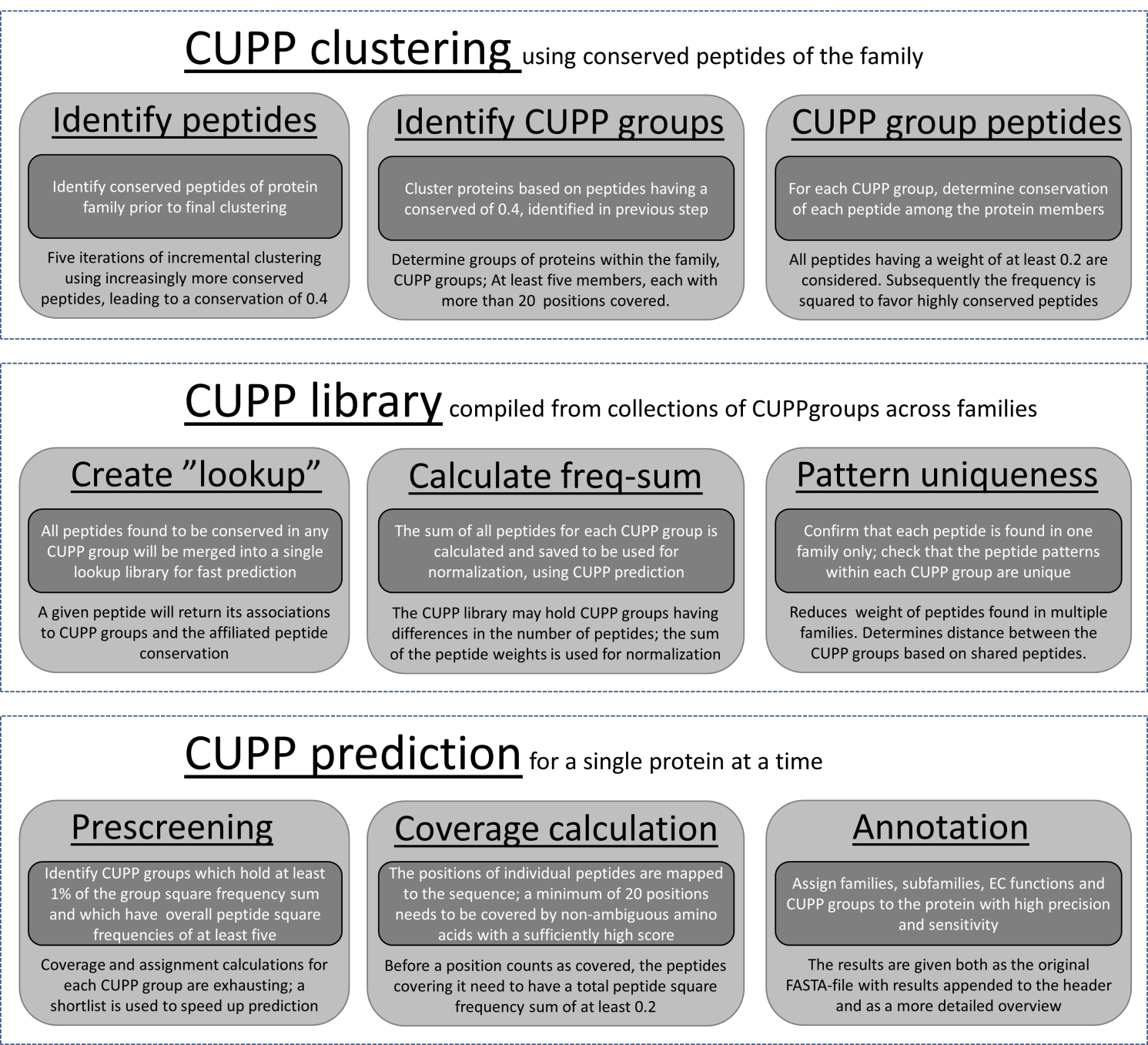


Figure S3 Flow diagram of the steps in the CUPP method, which start by taking a collection of proteins; than identify protein groups; and lastly find the conserved unique peptide patterns in each.

**Tables S1 - S2:**

**Table S1 Relative RAM requirements as a function of peptide length and number of ambiguos positions**

Table S1 The RAM requirements for holding the CUPP library during prediction is about 8.8 GB RAM. We assume there is a direct proportionality between the number of peptides considered conserved and the number of peptides in each protein. As an example the number of six amino acid long peptides (6x0) will be approximately the same as the length of the protein. However, approximately 28 times (eight choose two) more peptide combinations will need to be generated when eight amino acid long peptides are selected with two ambiguous amino acids (8x2) in each. Additionally, each peptide requires more characters to be stored (eight instead of six), resulting in 33% increase of RAM leading to a total increase 37.2 fold between 6x0 and 8x2.

**Table S2 GH30 CUPP group validation**

**Table S2** The precision and sensitivity of the predicted family, subfamily, function and CUPP group using the full length sequences of proteins assigned to a CUPP group. This allows inspection of the reliability of the individual CUPP groups. The 1153 proteins were placed in the 33 CUPP groups and could be predicted with a sensitivity of 0.996 whereas the sensitivity of all proteins (1298 non-redundant) of the family was 0.986. Precision [Preci] and sensitivity [Sensi] of prediction of family, subfamily, function and CUPP groups for each of the CUPP groups individually. Only proteins which have been assigned a CUPP group during clustering are shown. Proteins having EC function 3.2.1.8 or 3.2.1.136 were evaluated as 3.2.1.8&3.2.1.136 in the validation.

Table S3 - N-fold cross validation of GH30 using ten partitions

**Table S3** Summary of the precision and sensitivity of CUPP prediction for each of the 10 parts omitted, one by one during N-fold cross validation. The column “Total count” indicates the number of proteins having assigned family, subfamily or EC function, respectively. The family precision is one because no other families were included in the CUPP library at this time. The results of each partition of the N-fold cross validation have been mapped to the phylogenetic tree from Figure S2. Proteins having EC function 3.2.1.8 or 3.2.1.136 were evaluated as 3.2.1.8&3.2.1.136 in the validation. The proteins of the ten partitions may have up to 70% sequence identity according to CD-HIT.
